# Determination of lopinavir/ritonavir concentrations in four different oral solutions for the application of antiretroviral therapy in very young, HIV-1-infected children

**DOI:** 10.4102/sajhivmed.v22i1.1222

**Published:** 2021-05-11

**Authors:** Nils von Hentig, Carlo Angioni, Christoph Königs

**Affiliations:** 1 Internal Medicine II, HIVCENTER, Goethe University Hospital, Frankfurt, Germany; 2 Institute of Clinical Pharmacology, Goethe University Hospital, Frankfurt, Germany; 3 Department of Pediatrics and Adolescent Medicine, Goethe University Hospital, Frankfurt, Germany

Infants infected with HIV-1 are generally more affected by disease progression and mortality than older children.

Early initiation of antiretroviral therapy (ART) prevents progression to AIDS and death. However, therapy options for very young children are quite scarce, as many antiretrovirals are not approved for use in children younger than 3 years of age, and experience regarding the dosing, application, side effects and outcome is limited.^1,2,3,4,5,6^

An antiretroviral regimen containing lopinavir/ritonavir (LPV/r) in addition to two nucleoside reverse transcriptase inhibitors (NRTIs) is the main treatment option for HIV-1-infected infants in most African countries.^7,8^ The LPV/r combination is available as a meltrex tablet, in a fixed-dose combination added to NRTIs, or as a liquid formulation that is bodyweight-dose adjusted.^2,5,6,9,10,11,12,13^ The tablet formulation is not applicable for very young children because of their inability to swallow the tablet. Crushing of the LPV/r meltrex formulation is not recommended, and the liquid formulation remains the only alternative for very young patients. However, as the taste of the pure LPV/r liquid formulation is unpalatable, many parents seek alternative ways to give the medicine, including mixing the LPV/r oral solution with formula milk, for example, as part of the child’s nutrition. However, flocculation of the oral solution results, and no bioavailability data are available.

Following the report of parents administering the solution in formula milk, which was well tolerated by the child, and having observed a good virological response, lopinavir plasma levels were determined at the University of Frankfurt, following routine monitoring of the child on ART. The parents provided informed consent before the procedure. The standard pharmacokinetic testing protocol for the examination of antiretroviral plasma concentrations in small children allows sampling at the following time points: at 0, 1 and 2 h after the dosing interval. Each sample is < 1 mL whole blood in children < 2 years of age. The sample is then processed and analysed by means of liquid chromatography–tandem mass spectrometry (LC-MS/MS). The child was treated with the licensed dose (230 mg/m^2^) twice daily and surprisingly reached a c_min_ of 3330 ng/mL and c_max_ of 5610 ng/mL.

We therefore prepared several oral solutions by mixing the LPV/r liquid formulation together with glucose syrup, glucose syrup and ethanol, or formula milk, with and without ultrasound treatment, and tested these oral solutions for their taste and drug stability. Three samples each of (1) formula milk (10 mL Humana PRE containing 0.32 g fat, 0.76 g carbohydrate and 0.12 g protein), (2) ultrasound-treated formula milk (to improve solubility and inhibit flocculation), (3) glucose syrup, and (4) glucose syrup plus ethanol (chemistry-based decision to improve the solubility) were spiked with the same concentration of liquid LPV/r (4000/1000 ng/mL), and the concentrations of LPV/r were measured by means of validated LC-MS/MS.^14^ The accuracy of controls was 92% – 105% for lopinavir and 95% – 109% for ritonavir. The linearity of the calibration curve ranged from 100 ng/mL to 60 000 ng/mL.^15^ Three samples of each formulation were prepared in order to test the possible variability of sampling. The investigators controlled the taste by taking it orally and inhibiting flocculation by visual inspection; tasting was performed independently and then reported to each other.

The three samples of formula milk treated with ultrasound for 25 min and the three samples of untreated formula milk showed lopinavir concentrations ranging from 2450 ng/mL to 2590 ng/mL and from 2450 ng/mL to 2790 ng/mL, respectively. The samples dissolved in ethanol-glucose syrup and glucose syrup alone showed lopinavir concentrations of 3040 ng/mL – 3060 ng/mL and 1520 ng/mL – 4020 ng/mL, respectively (Table 1).

**TABLE 1:** Plasma concentrations of liquid lopinavir and ritonavir in samples, mixed with formula milk, glucose syrup or glucose syrup plus ethanol.

Oral solution	Sample	LPV concentration		RTV concentration	
		ng/mL	%	ng/mL	%
Formula milk*	A1	2590	64.75	761	76.1
	A2	2630	65.75	779	77.9
	A3	2450	61.25	754	75.4
Formula milk	B1	2740	68.50	847	84.7
	B2	2550	63.75	795	97.5
	B3	2450	61.25	736	73.6
Syrup	C1	4020	100.50	1070	107.0
	C2	3110	77.75	967	96.7
	C3	1520	38.00	477	47.7
Syrup + ethanol	D1	3040	76.00	984	98.4
	D2	3060	76.50	996	99.6
	D3	3060	76.50	979	97.9

LPV, lopinavir; RTV, ritonavir.

* , The sample was treated by ultrasound for 25 min. The recommended minimum concentration for lopinavir in children is > 1000 ng/mL.

The samples were spiked with lopinavir/ritonavir 4000/1000 ng/mL each and subsequently dissolved 1:100 for LC-MS/MS analysis.

It was found that LPV/r flocculated in formula milk, regardless of whether the formula milk was ultrasound treated, and was intolerably bitter. In contrast, the taste of the LPV/r solution was completely masked when mixed together with glucose syrup. In general, the LPV/r liquid formulation proved to be stable for at least 15 min when dissolved in either formula milk or glucose syrup.

The samples from the LPV/r liquid formulation and glucose syrup mixture showed a marked variation in the lopinavir concentrations, but the variable results were not attributable to any instability of the drug in the resulting solution. This variation can be explained by sampling errors, because it was nearly impossible to accurately pipette small quantities of this highly viscous solution. This problem was confirmed through the addition of 1 mL ethanol to the syrup solution, which resulted in improved solubility as well as the most accurate concentration measurements obtained in this case.

In general, LPV/r can be dissolved in glucose syrup with a moderate loss of stability. The taste of the solution was greatly improved, so this method of application could very well help young children swallow the medication more easily. Dissolving the antiretroviral liquid formulation in formula milk resulted in flocculation of the solution, lower LPV/r concentrations and an incredibly bitter taste.

Because this report is based on a small sample size, a more comprehensive study should be carried out in order to confirm these findings and assist children living with HIV and their parents with the administration of the liquid LPV/r formulation.
